# The Prevalence of Multiple Sclerosis in 3 US Communities

**Published:** 2009-12-15

**Authors:** Dhelia M. Williamson, Curtis W. Noonan, Judy P. Henry, Laurie Wagner, Robert Indian, Sharon G. Lynch, John S. Neuberger, Randolph Schiffer, Janine Trottier, Ruth Ann Marrie

**Affiliations:** Centers for Disease Control and Prevention; Agency for Toxic Substances and Disease Registry, Atlanta, Georgia; Texas Department of Health, Austin, Texas; Texas Department of Health, Austin, Texas; Ohio Department of Health, Columbus, Ohio; University of Kansas School of Medicine, Kansas City, Kansas; University of Kansas School of Medicine, Kansas City, Kansas; Texas Tech University Health Sciences Center, Lubbock, Texas; Lorain County General Health District, Elyria, Ohio; Cleveland Clinic, Cleveland, Ohio

## Abstract

**Introduction:**

We estimated the prevalence of multiple sclerosis (MS) in 3 large geographic areas in the southern, middle, and northern United States.

**Methods:**

The primary data source was medical records from office visits to private neurologists' practices or to neurology departments in tertiary care facilities during a 3-year period. Additional data sources included patient advocacy groups, nursing homes, and general practitioners.

**Results:**

Three-year US age-adjusted prevalence estimates for the study areas varied substantially. The prevalence was lowest (47.2 per 100,000 population) in the Texas study area (33°30′ north latitude), intermediate (86.3 per 100,000 population) in the Missouri study area (39°07′ north latitude), and highest (109.5 per 100,000 population) in the Ohio study area (41°24′ north latitude). The geographic differences remained strong after age-adjustment to the world standard population. The inverse association between UV light exposure and MS prevalence estimates was consistent with this observed latitude gradient. In all 3 areas, MS prevalence was highest among women, people aged 40 to 59 years, and non-Hispanics.

**Conclusion:**

These results provide necessary prevalence estimates for community cluster investigations and establish baseline estimates for future studies to evaluate temporal trends in disease prevalence.

## Introduction

Multiple sclerosis (MS) is an inflammatory demyelinating disease of unknown origin; it affects more than 1 million people worldwide (1) and disproportionately affects women and whites (2). Approximately 85% of affected people have a relapsing-remitting course, characterized by an unpredictable course of exacerbations and remissions (3). Ultimately, most patients become disabled and may or may not have superimposed relapses (secondary progressive MS) (3,4). Approximately 15% of patients have primary progressive MS, in which the condition worsens gradually from disease onset and is not associated with relapses (5).

The ability of a public health agency to enumerate cases and determine whether an excess of cases is present in a particular community is compromised by the lack of MS registries, the range of disease severity, which can affect ascertainment, and unknown background prevalence estimates. Prevalence estimates for MS vary from 58 to 95 per 100,000 population in the United States (6-8). In the past 25 years, prevalence studies of specific US locales have produced a range of estimates (9), up to 177 per 100,000 population in Olmstead County, Minnesota (10). Our goal was to determine MS prevalence estimates for 3 areas in the southern, middle, and northern United States to be used as regional comparisons for community-specific investigations of MS prevalence and as baseline estimates for studies of temporal trends in prevalence.

## Methods

### Study areas

The areas we studied were Lorain County, Ohio; the cities of Sugar Creek and Independence, Missouri; and 19 counties surrounding Lubbock, Texas. Lorain County (population 284,664) was included in this study because the community had expressed concerns about the frequency of MS in the city of Wellington (population 3,171) and the possible health risks of proximity to an iron foundry and plastics company. Results from a previous cluster investigation in Wellington identified 27 cases of MS for a crude point prevalence estimate of 651 per 100,000 persons, but this assessment was limited by the lack of appropriate US comparison prevalence estimates for Ohio (11).

Another community, Sugar Creek, expressed similar concerns about a perceived excess of MS cases and was near an oil refinery that operated in the area from 1904 to 1982 (12). Although refinery operations ceased in 1982, portions of the site are still used as a light-oil petroleum product marketing terminal, a pipeline facility, and an asphalt receiving and processing center. Because of the small size of Sugar Creek (population 9,915), the adjacent city of Independence (population 110,884) was included for comparison.

The 19-county area around Lubbock (population 424,916) was proposed to provide MS prevalence estimates for comparison with a previous MS cluster investigation conducted in El Paso, Texas (13). In all 3 previous cluster investigations, the lack of an appropriate comparison for MS prevalence estimates precluded an assessment of the true effect of the disease.

### Case ascertainment

The primary data source for case ascertainment was medical records from the offices of neurologists practicing in the study area or in contiguous areas or from the neurology departments of local hospitals. In Ohio, this included offices and hospitals in Lorain and Cuyahoga counties, including the Cleveland Clinic, 8 private neurologists, and 5 hospitals. In Missouri, this included offices and hospitals in Clay and Jackson counties, Missouri, and Johnson and Wyandotte counties, Kansas, including 12 private neurologists and 8 hospitals that provided neurologic care. In Texas, the Texas Tech University Medical System and 8 private neurologists provided neurologic specialty services and care for the study area.

Residence was determined by the address in the patient's medical record. Records were included if patients had an office visit from January 1, 1998, through December 31, 2000, and had the following International Classification of Disease, 9th Revision, codes or corresponding conditions: MS (340), other demyelinating diseases (341.8-341.9), transverse myelitis (323.9), and optic neuritis (377.3).

We used additional data sources to evaluate the completeness of case ascertainment, including patient advocacy groups (the National Multiple Sclerosis Society), nursing homes, general practitioners, and death certificates. Self-reports were not actively sought; however, if people with potential cases identified themselves to study personnel, they were asked to provide the name of their treating physician. Similarly, treating physicians were identified for any potential cases identified through MS advocacy groups or nursing homes. If not already included in the surveillance effort, all medical records that met the inclusion criteria were abstracted from the treating physician's office. This study was approved by institutional review boards in each study area and by the Centers for Disease Control and Prevention.

### Case verification

Trained abstractors, who were supervised by the project investigators and neurologists, used a standard form that included history of relapses, neurologic examination findings, and results of evoked potentials, cerebrospinal fluid examination, and magnetic resonance imaging of the brain and cervical spine. Abstractors also recorded sex, race/ethnicity, occupation, family history of MS, country/state of birth, treating physician's MS diagnosis, criteria used to determine diagnosis, and dates of symptom onset and diagnosis. Individual identifiers (name, address, and Social Security number) were recorded to ensure accurate case counts and to avoid duplicate counting from other sources.

The abstracted records of all patients with potential MS cases were evaluated by reviewing neurologists in each study area according to the Poser criteria (14). We chose the Poser criteria because they were the criteria in use during the period evaluated. Each case was classified as definite (clinical or laboratory supported) or probable (clinical or laboratory supported) MS. We also considered 2 additional disease classification categories. The category of presumptive MS was used for cases for which data were insufficient to satisfy clinical criteria but for which a diagnosis seemed correct after review, and the category of unknown was used when data were insufficient to determine the presence or absence of MS. If necessary to classify a specific case, the neurologist directed the abstractor to collect additional information. We used only definite and probable categories to calculate prevalence for this analysis.

### Exposure to UV radiation

To explore the geographic difference in MS prevalence estimates, we evaluated the differences in UV radiation exposure between study regions. Patterns of exposure to UV radiation are similar to patterns of distribution of MS in some areas, which suggests a possible contribution to MS risk (15,16). We used archived data from the National Weather Service's UV Index Forecast averaged for 1997 through 2001 for sites near our study areas (17). The UV Index Forecast is the scaled erythemal (skin reddening) dose rate integrated over the UVB and UVA spectral bands. The data were summarized according to World Health Organization categories from low to extreme. The UV data locations (and their approximate distance from the respective study areas) were Cleveland, Ohio (29 miles); Saint Louis, Missouri (249 miles); and Dallas, Texas (347 miles). Each of these surrogate locations was in the same state and at approximately the same latitude as its respective study area.

### Data analysis

We calculated age- and sex-specific period prevalence estimates by using the definite and probable MS cases ascertained from 1998 through 2000 as the numerator and the 2000 census counts for the study areas as the denominator. Overall prevalence estimates for each area were directly race-adjusted to the US 2000 race/ethnicity distribution and age-adjusted to the US 2000 and the world standard populations (18,19). The differences between strata-specific prevalence estimates were evaluated by assuming a Poisson distribution. We used SAS version 9.0 (SAS Institute, Inc, Cary, North Carolina) for statistical analyses. To evaluate latitude, regression analysis was used to estimate the increase in MS prevalence for a unit increase (degree) in latitude.

## Results

All practicing neurologists in the 3 study areas participated and provided medical records for review. Abstractors screened 1,434 medical records, and 670 records did not meet the screening criteria for date of office visit or place of residence. The remaining 764 records were abstracted and reviewed by the study neurologists to confirm MS. Of these, 608 (80%) were classified as definite or probable MS. A small number of patients (7 in Texas, 17 in Missouri, and 4 in Ohio) had symptoms that suggested MS, but data were insufficient to satisfy the clinical criteria. These cases were not included in the prevalence estimates. In Missouri, approximately 20% of reviewed records were captured from tertiary care facilities with MS specialization facilities, compared with 54% in Texas and 57% in Ohio.

Overall prevalence estimates for the study areas varied greatly; the prevalence was lowest in Texas (33°30′ north latitude), followed by Missouri (39°07′ north latitude), and highest in Ohio (41°24′ north latitude). Prevalence estimates changed slightly after age-adjustment to the US or world standard populations, but the monotonic prevalence gradient remained consistent with the increase in US age-adjusted prevalence per degree increase in latitude of 7.7 (95% confidence interval [CI], −2.1 to 17.5) per 100,000 population. For US age-adjusted estimates, the difference in prevalence per 100,000 population between Texas and Missouri was 39 (95% CI, 21-57), and the difference in prevalence per 100,000 between Missouri and Ohio was 23 (95% CI, 3-54).

Race-adjusted prevalence estimates for the 3 areas showed a pattern similar to the age-adjusted estimates, although the difference in race-adjusted prevalence between Missouri and Ohio was only 16 (95% CI, −2.6 to 35.1) per 100,000 (Table). Prevalence was higher in non-Hispanic whites than in other racial/ethnic groups. In Texas, the difference in prevalence estimates between non-Hispanic whites and Hispanics was 45 (95% CI, 34-56) per 100,000. In Ohio, the difference in prevalence estimates between these 2 groups was 43 (95% CI, 8-79) per 100,000. The prevalence was lower in non-Hispanic blacks than in non-Hispanic whites in Texas, a difference of 34 (95% CI, 14-54) per 100,000, but the prevalences in these groups were not significantly different in Ohio. Data from Missouri concerning prevalence in minority populations were too sparse to draw any conclusions, possibly because of the smaller size of this study population. The MS prevalence was much higher in women than in men (4.1, 3.9, and 2.8 times as high in women in Texas, Missouri, and Ohio, respectively) (Table).

When evaluating the UV data, 46% of days were classified as high to extreme UV exposure in Texas (the southernmost area with the lowest prevalence of MS), compared with 29% of days for Missouri (middle latitude) and 21% of days for Ohio (northern latitude) (*P* < .001, Cochran-Armitage test for trend).

## Discussion

In the United States, reported prevalence estimates for MS vary widely, which may reflect differences in ascertainment methods or in the underlying population structure (18,19). To the best of our knowledge, this is the first study to simultaneously estimate population-based MS prevalence in 3 US communities by using the same ascertainment and case-verification methods. Our prevalence estimates reflect the range of estimates previously observed and support the reported geographic heterogeneity of MS prevalence in the United States and elsewhere (20,21). The northernmost area in our study had the highest MS prevalence, and the southernmost area had one of the lowest MS prevalences observed in recent US studies. Although the Lubbock area had a high proportion of Hispanics (a group in which the prevalence of MS may be low or underreporting may be high) (22), the prevalence for non-Hispanic whites was only 56.0 per 100,000.

Higher prevalence estimates were observed among whites than among blacks in the Texas area, which is similar to national survey data in the United States (6-8). In the Ohio area, MS prevalence estimates were similar for non-Hispanic whites and non-Hispanic blacks. Race/ethnicity was undetermined for 45 (14%) of the MS patients in Ohio. Even if all of these patients were non-Hispanic whites, the resulting prevalence estimates per 100,000 would be 117.9 for non-Hispanic whites, compared with 90.9 for non-Hispanic blacks. These revised estimates do not significantly differ, and the absolute difference in prevalence estimates by race for this area was still much lower than that observed in the Texas area or in previous US studies. The reason for this difference between study areas is unknown, and unfortunately, racial heterogeneity in the third study area was insufficient to explore this issue further. The difference in prevalence estimates for Hispanics in Texas and Ohio is also noteworthy, but the reason is unclear.

A previous report noted that if incidence studies were age-adjusted to a common population, latitude was not associated with incidence (18). This analysis was limited to 22 incidence studies, none from the United States. Prevalence data were less affected by age adjustment; latitude associations remained after adjustment to the world population. These analyses included only prevalence studies from a single area in the United States at approximately 44° north latitude, and all studies were grouped by 10° latitude intervals. Furthermore, racial and ethnic differences in populations studied were not considered. Our results, even after age-adjustment, demonstrate a strong gradient within a small range in latitude, approximately 33° to 41° north latitude, with a prevalence ratio of 2.32 between the northern (Ohio) and southern (Texas) regions, similar to the relative risk of MS observed in US veterans according to where they lived when they joined the military (23). However, this study and others suggest that the latitude gradient in the United States and other parts of the world is not as steep as previously suspected. These studies also suggest an increasing risk of MS in nonwhites, which emphasizes that prevalence estimates must be adjusted for sex and race (20,24).

The geographic distribution of MS is interpreted by some researchers as reflecting differences in the distribution of genetically susceptible populations, as determined by racial and ethnic backgrounds (25,26). Our study areas showed a geographic gradient even when we restricted the analysis to non-Hispanic whites, but we could not characterize patient ancestry. Migration studies suggest an association between geography and MS risk (27,28), but unless migration patterns were differential between regions, this association would not explain our findings.

Differences in environmental risk could also influence geographic variation in MS prevalence, and some studies have postulated that UV exposure could influence this variation. The geographic gradient in MS prevalence in Australia correlates strongly with UV exposure (16). Similarly, in a US case-control study, high levels of residential and occupational sunlight exposure were inversely associated with risk of MS (15). Our data support this inverse association between UV exposure and MS prevalence, although the mechanism by which UV exposure may influence MS risk is unknown (16). UV exposure is only 1 of several factors that could explain the epidemiologic features of MS, but such questions cannot be answered with this ecologic analysis.

This project had several strengths. First, the application of consistent case-finding and case-verification method to 3 geographically distinct areas allowed us to evaluate the previously reported latitude gradient for MS. We captured age- and sex-specific prevalence estimates, which allows adjusted comparisons with MS prevalence studies worldwide. Second, we captured race/ethnicity data for 89% of cases, allowing for an evaluation of racial/ethnic differences in MS prevalence. Finally, medical records review usually provided sufficient data to verify MS diagnosis according to rigid clinical criteria. This cost- and time-effective approach to data gathering had high specificity for identifying MS cases.

Although our use of a rigid case definition enhanced specificity, it may have reduced sensitivity of ascertainment. Although abstracted records typically included extensive case histories, some borderline cases would have required a clinical visit for accurate diagnosis, and some records may have contained insufficient information if patients had recently changed physicians. For the 3 areas combined, however, less than 4% of reviewed records strongly suggested MS but data were insufficient to classify them as definite or probable. If the final case counts included all these records, the crude prevalence estimates per 100,000 would be 44 in Texas, 102 in Missouri, and 114 in Ohio.

Because the primary data source was neurologists' practices and neurology departments of tertiary care facilities, some cases were probably missed (29), particularly among the small proportion of patients with benign disease or relapsing-remitting MS in sustained remission. These patients may not regularly seek neurologic care and may not have been captured during our study period, and a longer study period may have captured less severe MS cases. Few cases were captured through general physicians because we did not actively pursue this source. We may have also missed MS patients who did not have access to the health care system. The sites differed with respect to the proportion of private neurology practices versus tertiary-care facilities as the source of case ascertainment, which could lead to differences in the completeness of case ascertainment. Regardless of differences in the medical communities serving the respective areas, however, both of these sources are considered the most appropriate data sources (29). Where estimates of MS prevalence exist for regions of similar latitude, our prevalence estimates are similar (29,30).

Our study established the feasibility of using a uniform method for case ascertainment in different geographic regions and demonstrated that the distribution of MS varies with respect to geography, sex, and race/ethnicity. It provides necessary background prevalence estimates for cluster investigations while establishing baseline estimates for future studies evaluating temporal trends in prevalence.

## Figures and Tables

**Table:** Cases of Multiple Sclerosis and Respective Strata-Specific and Adjusted Prevalence Estimates for 3 Areas, 1998-2000

Characteristic	Texas (Lubbock and 19-County Surrounding Area)			Missouri (Independence and Sugar Creek)			Ohio (Lorain County)		

	No. of Cases^a^	Population	Prevalence per 100,000 (95% CI)	No. of Cases^a^	Population	Prevalence per 100,000 (95% CI)	No. of Cases^a^	Population	Prevalence per 100,000 (95% CI)
**Sex**									
Women	147	214,235	68.6 (58.0-80.6)	86	62,870	136.8 (109.3-169.2)	237	145,017	163.4 (142.2-184.6)
Men	35	210,681	16.6 (11.6-23.1)	20	57,929	34.5 (21.3-53.2)	83	139,647	59.4 (46.4-72.4)
**Age, y**									
<30	19	201,420	9.4 (5.7-14.7)	4	47,683	8.4 (2.4-21.7)	10	116,254	8.6 (4.0-15.9)
30-39	33	57,282	57.6 (39.7-80.9)	13	17,244	75.4 (40.3-129.1)	34	42,485	80.0 (52.4-107.6)
40-49	59	57,239	103.1 (78.5-132.9)	40	18,172	220.1 (156.9-299.7)	97	45,663	212.4 (169.3-255.5)
50-59	49	40,869	119.9 (88.7-158.5)	34	14,078	241.5 (167.5-337.0)	102	33,274	306.5 (245.8-367.2)
60-69	17	30,676	55.4 (32.3-88.7)	11	10,247	107.3 (53.3-192.3)	40	21,088	189.7 (129.7-249.7)
≥70	4	37,430	10.7 (2.9-27.4)	4	13,375	29.9 (7.9-76.8)	37	25,900	142.9 (95.9-189.9)
Unknown	1	NC	NC	0	NC	NC	0	NC	NC
**Race/ethnicity**									
Hispanic	16	142,448	11.2 (6.4-18.2)	0	2,450	NC	11	19,642	56.0 (21.3-90.7)
Non-Hispanic white	140	249,882	56.0 (47.1-66.1)	104	108,957	95.5 (77.9-115.6)	242	243,388	99.4 (86.6-112.2)
Non-Hispanic black	6	27,173	22.1 (8.1-48.1)	1	2,984	NC	22	24,196	90.9 (52.1-129.7)
Other/unknown	20	NC	NC	1	6,408	NC	45	NC	NC
**Crude overall**	182	424,916	42.8 (36.8-49.5)	106	120,799	87.7 (71.6-106.4)	320	284,664	112.4 (99.8-125.0)
**Race-adjusted^b^**	162	NC	44.2 (37.2-51.2)	105	NC	72.6 (57.2-88.0)	275	NC	88.9 (78.1-99.7)
**US age-adjusted^c^**	182	NC	47.2 (40.3-54.1)	106	NC	86.3 (69.8-102.8)	320	NC	109.5 (97.5-121.6)
**World age-adjusted^d^**	182	NC	39.9 (34.0-45.7)	106	NC	70.6 (56.9-84.3)	320	NC	86.5 (76.8-96.2)

Abbreviations: CI, confidence interval; NC, not calculable.

a Cases include diagnoses of definite or probable MS according to the Poser 1983 criteria (14).

b Directly race-adjusted to the 2000 US population. Cases with missing/unknown data on race/ethnicity (20 in Texas, 1 in Missouri, and 45 in Ohio) were not included in specific strata for race/ethnicity prevalence.

c Directly age-adjusted to the 2000 US population.

d Directly age-adjusted to the 2000 world population. To calculate age-adjusted prevalence, cases with missing data on age (1 in Texas and 3 in Ohio) were assumed to be in the age group with highest prevalence, 50-59 years.
